# Maternal Ingestion of *Ipomoea carnea*: Effects on Goat-Kid Bonding and Behavior

**DOI:** 10.3390/toxins8030074

**Published:** 2016-03-16

**Authors:** André T. Gotardo, James A. Pfister, Paulo C. F. Raspantini, Silvana L. Górniak

**Affiliations:** 1 Research Center of Veterinary Toxicology (CEPTOX), Department of Pathology, School of Veterinary Medicine and Animal Science, University of São Paulo, Pirassununga 13635-900, Brazil; andregotardo@gmail.com (A.T.G.); pcraspantini@gmail.com (P.C.F.R.); 2 United States Department of Agriculture, Agricultural Research Service (USDA-ARS), Poisonous Plant Research Laboratory, 1150 E. 1400 N., Logan, UT 84341, USA; jamesapfister@gmail.com

**Keywords:** swainsonine, reproductive toxicology, neuroteratology, goats, morning glory, *I. carnea*

## Abstract

*Ipomoea carnea* is a toxic plant found in Brazil and other tropical and subtropical countries and often causes poisoning of livestock. The plant contains the alkaloids swainsonine and calystegines, which inhibit key cellular enzymes and cause systematic cell death. This study evaluated the behavioral effects of prenatal ingestion of this plant on dams and their kids. Twenty-four pregnant goats were randomly allocated into four treatment groups and received the following doses (g/kg BW) of fresh *I. carnea*: 0 (control group), 1.0 (IC1), 3.0 (IC3), and 5.0 (IC5) from day 27 of gestation until parturition. Dam and kid bonding and behavior were evaluated by several tests, immediately after birth until six weeks of age. Dams from IC3 and IC5 groups spent less time paying attention to the newborn. There was a lack of maternal-infant bonding due to *I. carnea* intoxication. Kids from treated dams had difficulty in standing, suckling, and in recognizing their mother hours after birth. *I. carnea* can also compromise the kids’ ability to learn and to retain spatial memory. We suggest that kids from pregnant goats given *I. carnea* during gestation have significant behavioral alterations and developmental delays that may compromise their survival.

## 1. Introduction

*Ipomoea carnea* (*I. carnea*) is a shrubby plant in the Convolvulaceae family which originated in tropical America and the Caribbean, but is now found in tropical and subtropical areas worldwide including South and North America, Africa, Australia, and Asia [1,2].

Livestock that chronically ingest this plant suffer from extensive losses including death, abortion, and other reproductive and physiological dysfunctions, that have been reported in different countries such as Brazil [3,4,5,6], Peru [7], and Mozambique [8]. The intoxication typically occurs during the dry season when forage is scarce; cattle, sheep, and goats are the primary affected species [2,5].

The toxins in *I. carnea* are the indolizidine alkaloid swainsonine and the nortropane alkaloids calystegines. Swainsonine has been demonstrated to be produced not by the plant, but by fungi living in or on the plants. Swainsonine, but not calystegines, are produced by fungi in the order Chaetothyriales that live epiphytically with *I. carnea* [9]. Swainsonine is a powerful inhibitor of lysosomal α-mannosidase and Golgi mannosidase II [8,10,11,12]. Swainsonine has also been isolated from other plants, such as *Swainsona canescens* in Australia [13], *Astragalus* and *Oxytropis* species, often termed “locoweeds” in North America [14], and *Sida carpinifolia* and *Turbina cordata* in Brazil [6]. Calystegines inhibit lysosomal α-galactosidase and β-glycosidase [15], and we verified in rats, that these alkaloids potentiate the lesions produced by swansonine [16]; however, it is unknown how the nortropane alkaloids contribute to the toxicity of *I. carnea*, particularly in a ruminant model.

The inhibition of lysosomal α-mannosidase results in lysosomal accumulation of incompletely processed oligosaccharides, vacuolation, and cellular death [17]. In goats such damage is especially severe in the CNS [3,8,18,19,20,21]. This vacuolar degeneration can be seen in other organs such as the thyroid, liver, pancreas, and kidneys [22,23].

Swainsonine’s inhibition of Golgi mannosidase II leads to changes in the synthesis, processing, and transport of glycoproteins, which causes dysfunction in hormones and membrane receptors [24] and leads to alterations in endocrine [25,26,27] and reproductive functions [27,28].

In goats, the signs of *I. carnea* intoxication are mostly of nervous origin; clinical signs include depression, a staggering gait, muscle tremors, ataxia, nervousness, and weight loss [5,21,23,29]. Also, we verified that the administration of 7.5 g/kg BW of *I. carnea* leaves to pregnant goats results in fetal death, abortion, stillbirths, cytoplasmic vacuolation in the fetal CNS, and structural and functional changes in the offspring [21].

Many studies have shown the teratogenic effects of pesticide residues in ruminants [30], as well as teratogenicity from toxins such as cyanide [31,32,33] and some classes of alkaloids [21,34,35,36,37,38]. The evaluation of the teratogenic potential of toxicants in ruminants has the potential to improve animal production and thus provide numerous economic benefits to livestock producers [38]. Protocols for developmental toxicology assessment established by regulatory agencies have received increasing importance and attention. It is known that changes in the fetus may be both structural and functional and caused at any time between fertilization and post-natal maturation [39]. Thus, these protocols are becoming more refined and specific, detecting more subtle changes that previously went unnoticed by classical protocols, such as bone and visceral evaluations [40]. In this sense, we have sought to refine an earlier proposed model for toxicity risk assessment in ruminants that had been developed in this laboratory [21,29,32,41]. The objective of this study was to assess the behavior of the offspring from mothers treated with *I. carnea* during pregnancy in order to refine current knowledge about neonate responses.

## 2. Results

### 2.1. Swainsonine and Calystegine Concentration

*I. carnea* leaves contained 0.14% swainsonine based on their dry weight. Thus, it was calculated that pregnant goats from the different groups ingested the following daily doses of swainsonine, in mg/kg BW: IC0 = 0.0; IC1 = 0.27; IC3 = 0.81; IC5 = 1.35. The calystegine concentrations in the plant were 0.03%, 0.07%, and 0.03% for calystegines B1, B2, and C1, respectively.

### 2.2. Clinical Evaluation’ Body Weight Gain’ and Birth Weight

None of the pregnant females in the different groups showed any clinical changes during the experimental period. No dystocia was observed. No significant changes (Table 1, *p* > 0.05) were detected in weight gain during pregnancy. In the IC3 group, there was one aborted fetus on day 139 of gestation and in the IC5 group one aborted fetus on day 112 of gestation (Table 1). The body weight of the kids at birth was unaffected by treatment (Table 1, *p* > 0.05).

### 2.3. Maternal Behavior Two Hours Postpartum

Dams from the IC3 and IC5 groups spent less time (min) attending to their kids in either the first and second hour after parturition compared with controls (*p* < 0.05; Table 2). Treated dams from IC3 and IC5 reduced their frequency of sniffing/licking their kids during the first and third period (0–15 and 30–60 min postpartum respectively) compared with controls (*p* < 0.05; Table 2). During the second time period (15–30 min postpartum) IC5 dams had a lower frequency of sniffing/licking their kids than did control dams (*p* < 0.05; Table 2).

### 2.4. Kid BehaviorTwo Hours Postpartum

Kids from the IC3 and IC5 groups differed from controls in the latency from birth to first attempt to stand (*p* < 0.05; Table 3). Only kids from the IC5 group differed from controls in the latency to successfully stand (*p* < 0.05; Table 3). Kids from the IC3 and IC5 groups spent less time standing than controls (*p* < 0.05; Table 3). All control kids were able to suckle successfully. In the IC1 group, five of six kids suckled successfully compared to 4/6 for the IC3 group and 3/8 for the IC5 group.

Kid behavior in first 0–15 min postpartum. In the first 15 min, kids from the IC3 and IC5 groups had a numerically higher frequency of crawling behavior and reduced frequency of attempts to stand compared with control kids (*p* < 0.05; Table 4). Numerically, control kids had increased episodes of standing up, nuzzling either the dam’s front half or rear half, and walking to the dam compared with kids from treated dams (*p* < 0.05; Table 4).

Kid behavior 15–30 min postpartum. Again in this period kids from the IC1, IC3, and IC5 groups had reduced frequencies of nuzzling their dam’s front and rear halves, and suckling and walking to their dam compared to kids from the control group (*p* < 0.05; Table 4).

Kid behavior 30–60 min postpartum. During the 30 to 60 min period, IC5 kids had a lower frequency of nuzzling their dam’s rear half compared to control kids (*p* < 0.05; Table 4). Furthermore control kids had higher frequencies of suckling and walking to their dam compared with IC1, IC3 and IC5 kids (*p* < 0.05; Table 4).

Kid behavior 60–120 min postpartum. In this period, control kids had a higher frequency of walking to their dam compared to kids from treated dams (*p* < 0.05; Table 4).

### 2.5. Dam-Alien Goat Discrimination Test

Kids from groups IC1, IC3, and IC5 made many more errors in choosing their own dam in the discrimination test than did control kids. Choices by kids from treated dams did not differ from randomness, whereas control kids did not choose randomly (*p* < 0.001; Table 5). In the first run, control kids were faster than kids from the IC1, IC3, and IC5 groups in time to exit the start box and time to arrive at the dams (*p* < 0.05; Table 5). In the second run, control kids differed only from IC3 and IC5 kids in time to exit the start box and time to arrive (*p* < 0.05; Table 5).

### 2.6. Kids Tests of Movement

There was no treatment × day × run interaction for leaving the start point of the maze and for arrival at the end (*p* > 0.05), therefore results are shown only for days in Table 6. During the test on days 2 and 4, control kids were faster to leave the starting point and arrive at the end near their mother than kids from the IC3 and IC5 groups (*p* < 0.05; Table 6). On day 6, control kids left the starting point and arrived at the end more quickly than IC3 kids (*p* < 0.05; Table 6).

### 2.7. Hebb-Williams Mazes

Kids from the IC5 group made a greater total number of errors (*i.e.*, sum of errors for weeks 6, 8, and 10 combined) in the Hebb-Williams configuration A and C than kids from control group (*p* < 0.05; Table 7). There was no treatment × day × run interaction for leaving the start point of the maze and for arrival at the end (*p* > 0.05), therefore results are shown only for days in Table 8.

Kids performance in Hebb-Williams maze A. During the test in postpartum week 2, control kids were faster than IC5 kids in leaving the start point and quicker to reach the end of the maze compared with kids from IC1, IC3, and IC5 groups (*p* < 0.05; Table 8). In postpartum week 4, control kids differed from kids from the IC1 group in leaving the start point (*p* < 0.05; Table 8).

Kids performance in Hebb-Williams maze B. In postpartum week 2, control kids arrived more quickly at the end of the maze than IC5 kids (*p* < 0.05; Table 8). In postpartum week 6, control kids left the starting point and reached the end of the maze faster than IC5 kids (*p* < 0.05; Table 8).

Kids performance in Hebb-Williams C. Control kids were faster than IC5 kids in leaving the start point in postpartum week 2 and in reaching the end of the maze in postpartum week 6 (*p* < 0.05; Table 8).

Kids performance in Hebb-Williams D. During the test in postpartum weeks 2 and 4, kids from the control group were quicker than kids from IC3 and IC5 groups in their arrival time (*p* < 0.05; Table 8). In postpartum week 6, control kids were faster than IC1 and IC5 kids in arrival time and faster than IC5 kids in leaving the starting point (*p* < 0.05; Table 8).

## 3. Discussion

Swainsonine, but not calystegines, are produced by fungi that live epiphytically with *I. carnea*. *Alternaria* subs. *Undifilum* produces swainsonine while living endophytically in *Swainsona canescens* [42], *Astragalus* sp. [43], and *Oxytropis* sp. [44]. Feeding rats with *Alternaria* subs. *Undifilum* induced clinical signs of locoism [45]. The contribution of calystegines, if any, to the toxicity of *I. carnea* has not been well established [16]. There is no evidence that *I. carnea* contains ergot-type alkaloids, nor the encoding genes for *Periglandula* fungi often present in Convolvulaceous plants (D. Cook, personal communication).

Studies on the teratogenic effects of plant toxins and understanding the relationship between chemical structure and activity may serve as a model for biomedical research and accelerate the discovery of new techniques and products for treating human and animal diseases [38]. Studies have shown the teratogenic effects of different alkaloids both for humans [46] and for animals [38]. Recently Welch *et al.* [47] showed that anabasine, an alkaloid from *Nicotiana glauca*’ is a potent teratogenic compound for goats, causing skeletal contracture malformations and cleft palate. Similarly, our studies verified that kids whose mothers ingested *I. carnea* during pregnancy may have structural changes such as arthrogryposis and retrognathia, and functional alterations such as cytoplasmic vacuolation, primarily in the CNS, but also in the liver and kidneys caused by the indolizidine alkaloid swainsonine [21]. Therefore, to better understand the consequences of vacuolar degeneration in the CNS of neonates, we decided to evaluate maternal and neonate behavior.

A prior study conducted in our laboratory showed that pregnant goats that ingested leaves of *I. carnea* at a dose of 7.5 g/kg BW had neurological signs of intoxication and also early fetal death, stillbirths, or abortion [21]. Therefore, in this study we decided to use lower daily doses of plant (1, 3, and 5 g/kg BW leaves of *I. carnea*), aiming to reduce the toxic effects of the plant on mothers and the possible indirect effect on the fetus [48]. Clinical evaluation revealed that we succeeded in this goal since pregnant females from different groups did not show any clinical signs of poisoning by *I. carnea*, and the body weight gain of pregnant females from experimental groups was similar to the control group. The correctness of the doses used was also shown by the fact that we did not observe any malformations or low birth weight in kids from any experimental groups.

Although no females showed overt clinical signs of poisoning from *I. carnea*, one goat from the IC3 group and one goat from the IC5 group aborted in late pregnancy. Other causes of abortion in goats, such as leptospirosis, brucellosis, mycoplasmosis, and toxoplasmosis were negative. Swainsonine has been shown to be an abortifacient compound in previous work with *I. carnea* [49] and locoweeds [35,50].

Behavioral results indicate that goats fed the higher doses of *I. carnea* (from IC3 and IC5 groups) spent less time paying attention to the newborn in the two hours after birth, as they sniffed and licked the kid with less frequency than did controls. Maternal behavior is a critical variable that influences the survival of the neonate [51], particularly under extensive grazing conditions. The maternal behaviors of sniffing and licking the kid immediately after birth are crucial for clearing the head and nostrils, thus stimulating respiration [51,52], blood circulation [53], and inducing the neonate to stand up and suckle [54]. Further, such actions aid in the formation of an exclusive olfactory memory in the mother for her own neonates [55]. Maternal inattention to the newborn after birth may result in the loss of maternal-infant bonding that is formed immediately after birth that allows mutual recognition during lactation [56,57]. Therefore, the decrease in maternal care observed in this study may result in a high neonatal mortality. Similarly, Pfister *et al.* [36] showed that fetal maternal-infant bonding in ewes can be impaired by ingestion of locoweed during pregnancy such that subsequent lamb survival depended on human intervention.

An important result in this study was the observation of low vigor in kids from mothers of the different groups treated with *I. carnea*. Offspring vigor influences the survival of the neonate [51] and may be responsible for the lack of bonding [58]. After birth, lambs pass through behavioral events directed towards standing, udder seeking, and sucking [55]. We verified that in the two hours after birth, kids from intoxicated mothers took longer to attempt to stand up and to stand, consequently they were much less mobile than were control kids. Standing and subsequent movement while teat-seeking reduces heat loss to the ground, so helping the lamb to maintain body temperature, and provides the easiest way to find the udder [55]. In fact, we observed that the number of kids able to suckle in two hours after birth decreased in a dose-dependent manner (control, 9/9; IC1, 5/6; IC3, 4/6; and IC5, 3/8).

Suckling quickly provides essential nutrients, particularly to sustain thermoregulation, and immunoglobulins to provide passive immunity [55,59]. In this sense, the faster the lamb accomplishes neonatal behaviors like standing and suckling, the higher the probability of survival [60]. These observations indicate that the alterations in behavior are potentially life-threatening to kids, as kids from treated dams were generally not able to stand, or were very slow to stand and suckle normally.

The high frequency with which kids from control group walked toward their dams in the first two hours postpartum was an excellent indicator of the kids’ ability to stand, walk, and suckle their dams. The higher vigor and desire to suckle in control kids during the first hour of life were confirmed by the increased frequency with which they nuzzled their dams front and rear halves and then suckled effectively. In the present study, we verified that control kids spent much of their time resting after suckling, during the final hour of observation; therefore major differences in behavior among control kids and kids from treated dams were not observed in this period.

Gotardo *et al.* [21] showed that the lesions in the CNS of kids whose mothers ate *I. carnea* during gestation were characterized by vacuolization and loss of Purkinje cells. Therefore, it can be inferred that the changes observed in kids from treated dams during the first two hours of life are directly related to impaired CNS functions from maternal exposure and fetotoxicity [61,62].

Kids from treated dams that were not able to suckle were given human assistance to suckle at the conclusion of the first portion of the study. For this, their dams were restrained by hand, and the kid was attached to the teat, and all suckled at that point. Thus, the treated kids, although typically low in vigor, all survived and were available to participate in the next tests(*i.e.*, Dam-alien goat discrimination test, kids tests of movements, and Hebb-Williams mazes).

The results of the dam-alien goat discrimination test and kid tests of movement confirmed the hypothesis of disrupted maternal-infant bonding in treated animals. Thus, these tests demonstrated the low vigor of newborns exposed in utero to *I. carnea*. Hence, kids from treated mothers were slower to traverse a maze to reach their dams, slower to reach their mothers around a barrier and were also unable to discriminate their dam from an alien goat at 12 h postpartum.

Goats have determinant behaviors for the survival of the newborn, and are an example of “hider” behavior at parturition [63]. Mothers and their young spent most of their time apart from one another during the infant’s first 6–8 weeks following birth [64]. So while the mothers graze, their kid(s) are hidden waiting for their return. This strategy is used to avoid predators; however, the success of this procedure depends on the kid’s ability to discriminate their mother, not confusing, for example, the approach of their dam with a predator. Furthermore, if a kid approaches the wrong dam, the alien dam often butts the young goat with considerable force [65] which could cause serious injury in weak kids and further complicate their ability to survive.

There are many procedures that can be used to evaluate learning in different animal species [66], but few studies have examined the influence of plant toxins on learning in livestock. However, such work has been done in horses [67], sheep [68], and cattle [69]. Lee *et al.* [68] showed that sheep under the effect of scopolamine had learning deficits in maze tests. Other studies with mazes showed cognition changes in sheep after exposure to toxicants [70]. Therefore, we proposed in this study to perform the assessment of learning using basically four mazes or closed fields (“Hebb-Williams A, B, C and D”), as proposed by Hebb-Williams [71] in which kids were tested at two, four, and six weeks of age.

Data from evaluations with Hebb-Williams mazes showed that kids from experimental groups showed a higher latency time to complete the different mazes until the sixth week of life, and goats from the IC5 group committed a greater number of errors in two mazes. Therefore, kids exposed to the *I. carnea* during pregnancy appeared to have reduced spatial awareness when in the maze and were less able to learn how to complete the maze. Sheep tested in mazes can retain spatial memories for many days [68], and control kids in our study also appeared to retain memories of the maze since their performance improved every week in all mazes; however, we did not observe this response so clearly in the performances of kids from groups of *I. carnea*-treated mothers. This result is similar to a study with sheep and swainsonine which showed that lambs exposed in utero did not improve their maze performance over the course of several days postpartum [36]. Under field conditions these results may have serious implications for growth and development. Kids from treated dams may be handicapped in their ability to learn from their dam and cohorts about foraging [72], location of food and water, and other aspects important for survival [73].

Little work has been done to characterize the effect of other swainsonine-containing plants such as locoweeds in goats. Furlan *et al.* [74] used a high swainsonine dose of 8 mg/kg BW from *Astragalus lentiginosus*, and pregnant goats became severely intoxicated in 10 days. Fetal death was observed in all locoweed-treated goats between 10 and 20 days after the beginning of locoweed treatment. Stegelmeier (unpublished personal communication) observed clinical signs of locoweed poisoning using a swainsonine dose of 1.5 mg/kg BW from *Astragalus lentiginosus* dosed for 45 days to Spanish goats. A dose of 1.5 mg/kg BW from *Astragalus lentiginosus* is higher than the highest swainsonine dose (1.35 mg/kg BW) used in this *I. carnea* study. Further studies are needed to characterize the relative contribution, if any, of calystegines to intoxication in goats from *I. carnea* compared to swainsonine alone [75]. In a rat model, calystegines alone added little to the neurotoxic syndrome [16], but rats are not a good model for glycosidase inhibitors. Studies are ongoing to compare the clinical signs and histopathology of swainsonine-containing locoweeds (e.g., *Astragalus lentiginosus* that contain no calystegines) and *I. carnea* at equal doses of swainsonine in a goat model.

## 4. Conclusions

The economic cost of livestock exposure to swainsonine-containing plants such as *I. carnea* may be high. In addition to the negative impacts on growing animals [23] and male and female reproduction [21,27] this study showed that the maternal responsiveness of goats was negatively affected by *I. carnea* ingestion. Dams that ingest *I. carnea* during gestation are less dedicated to newborns after birth, compromising the survival of the offspring. Kids exposed in utero to *I. carnea* showed low vigor at birth that would lead to poor kid survival, and later developmental delay if the kid survived. Kids from treated dams had difficulty in standing, suckling, and in recognizing their mother hours after birth. Maternal exposure to *I. carnea* can also compromise the kid’s ability to learn and to retain spatial memory.

## 5. Materials and Methods

This study was conducted at the University of São Paulo (USP) Experimental Station, Pirassununga, Sao Paulo state, Brazil (21°58’ S, 47°27’ W). The procedures were approved by the USP Animal Ethics Committee (protocol number 1402/2008, 27 May 2008), and all animal care and handling was performed by experienced personnel under veterinary supervision.

### 5.1. Plant Collection and Swainsonine Content

A plantation at the USP Experiment Station was the source of freshly harvested *I. carnea* leaves. The leaves were harvested daily, chopped in a feed mill, and immediately offered to individual female goats; all treated animals readily ate all offered *I. carnea* during the exposure period.

Samples from 20 plants in different areas of the plantation (1997 m²) were harvested at the beginning of the study and dried and ground (1 mm screen). The swainsonine concentration was determined on a composite of these samples by reversed phase high-performance liquid chromatography coupled with atmospheric pressure chemical ionization tandem mass spectrometry (LC-MS^2^ (Finnigan LCQ ITE, Mundelein, IL, USA)). The quantitation limit (*i.e.*, detection limit) was estimated to be 0.001% swainsonine by weight in dry plant material [76].The plants in the plantation were mature, and swainsonine concentrations in mature plants do not typically change greatly over time until senescence [77].

### 5.2. Animals’ Feeding’ and Experimental Design

Twenty four pregnant female goats (approximately 15 months of age) of the Alpine breed with an average weight of 35.0 ± 1.8 kg were used in this study. Based on antibody detection and mycoplasma culture, all females were determined to be negative for reproductive diseases caused by brucellosis, toxoplasmosis, leptospirosis, and mycoplasmosis. Breeding was synchronized using standard methods with vaginal pessaries, and all does were bred twice by the same fertile male of the same breed. The day of breeding was defined as day 1 of gestation, and the pregnancies were confirmed by an ultrasound (US) apparatus (Scanner 100 Vet, Pie Medical Lineal probe, 5.0/7.0 MHz, Pie Medical, Maastricht, The Netherlands) on day 27. Following this confirmation, the goats began to ingest the toxic plant.

Pregnant goats were randomly allocated into four treatment groups and given the following doses of *I. carnea*, in g per kg of body weight (g/kg BW) of *I. carnea*: 0 (control group, *n* = 6), 1.0 (IC1 group, *n* = 6), 3.0 (IC3 group, *n* = 6), and 5.0 (IC5 group, *n* = 6). Fresh *I. carnea* contained 19.3% dry matter, thus the groups IC1, IC3, and IC5 consumed daily 0.19, 0.58, and 0.96 g dry *I. carnea*/kg body weight, respectively. The treatment animals were provided with freshly harvested and chopped (2.5 cm screen) *I. carnea* from the 27th day of gestation to parturition (the 150th day of pregnancy approximately). The period of embryo implantation in goats begins on the 17th day and ends on day 23 post coitus [78]. Thus the females began to receive *I. carnea* after implantation during organogenesis. Each animal was provided with a commercial supplement to their diet (400 g/day): ground corn (60.6% on DM basis) and soybean extract (36%) with 3.4% mineral salt. Additionally, after the animals had consumed the commercial ration, and *I. carnea* for the treated animals, they were given access to chopped sugar cane residue (*Sacharum officinarum* L.) that was sufficient for overnight feeding *ad libitum*. Residual sugar cane was removed before feeding commenced the next day. Fresh water was available *ad libitum*. The animals were fed individually according to their assignment to treatments by securing them at the feeder to guarantee ingestion of the proper amount of *I. carnea*, but they were housed together in an open-air barn with a raised wooden floor, as is typical in Brazil. During gestation, the dams were clinically evaluated weekly and weighed every 15 days.

Pregnant goats were observed closely as their parturition date approached, and personnel were on hand when they gave birth. Immediately after birth, kid weight and sex were recorded. Each kid was then examined carefully for gross abnormalities [79].

### 5.3. Behavior Tests

Dam and kid bonding and behavior were evaluated immediately after birth to six weeks of age with several tests.

#### 5.3.1. Two Hour Postpartum Behavior

After birth, dams were given 5 min to bond with their offspring [80], and then both were moved to a separate pen for a 2 h observation period. In multiple births, the dam was not moved until the second kid was born, which typically occurred within a few min of the first birth. The 5 × 3 m pen had a 1 m grid marked on the concrete floor for reference. This 2 h period was videotaped and behavioral observations as noted below were made from these taped sessions. The postpartum session was divided into the following time periods: 0–15, 15–30, 30–60, and 60–120 min. Kid behaviors were recorded for latency to first occurrence of attempt to stand and successful to stand. Also were recorded time standing and the following frequencies: (1) crawl: paddling movement of the legs as if attempting to stand, but unable to get legs under the body and not able to lift body off the ground; (2) head up: attempt by the kid to lift its head off the ground any distance; (3) attempt to stand: legs under body and able to lift body off the ground any distance; (4) stand up: successful attempt to stand on all legs regardless of the length of time; (5) nuzzle dam’s front half: any nuzzling on the front half of the dam; (6) nuzzle dam’s rear half: any nuzzling on the rear half of the dam; (7) suckle: successful grasping of the dam’s teat with suckling for any length of time; each successful grasp of a teat with suckling was considered a bout of suckling; (8) walk to dam; and (9) walk away from dam. Dam’s behavior was also recorded during these periods. The single variable was: time spent by dams attending to their kid (*i.e.*, sniffing/licking kids).

#### 5.3.2. Dam-Alien Goat Discrimination Test

**Figure 1:**
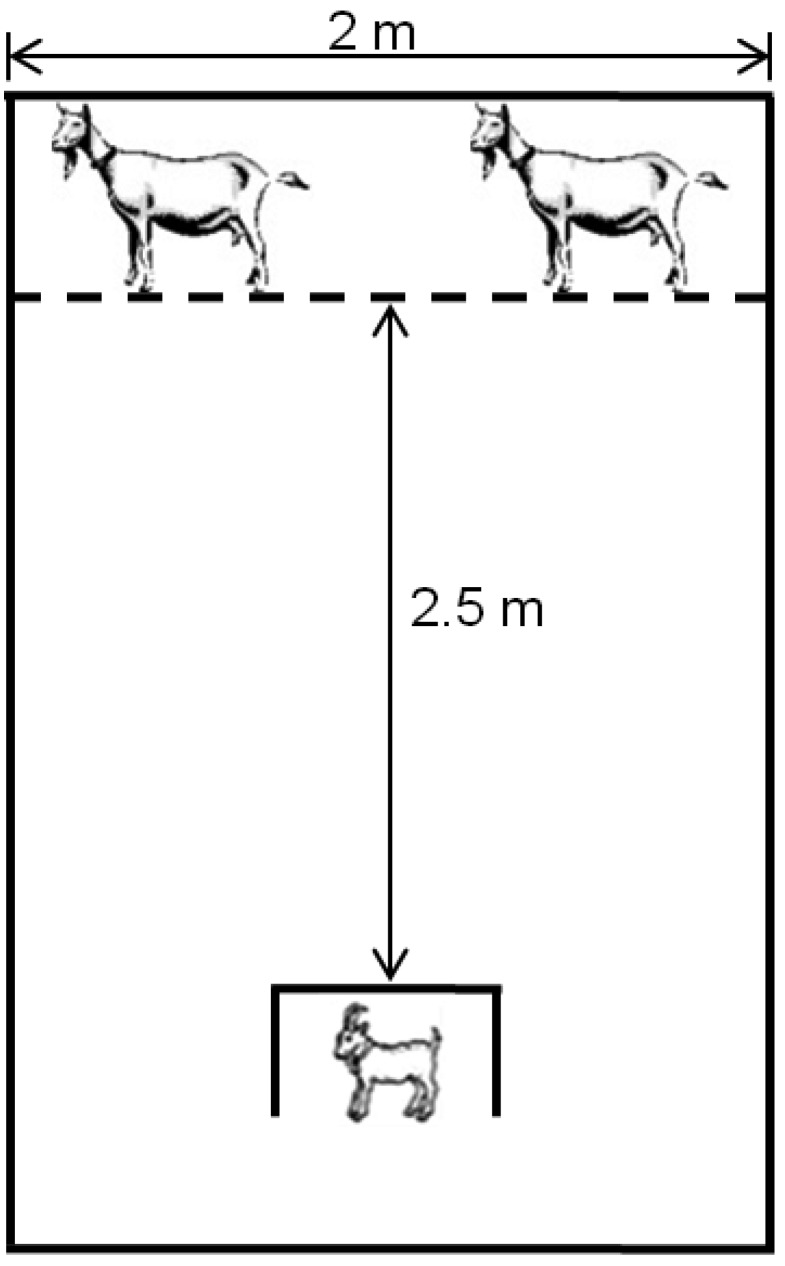
Schematic diagram of a maze used to test kid discrimination between its mother and an alien dam. The barrier represented by the dotted line was a wire-mesh panel through which kids could contact dams. Kids were tested at 12 h after birth, except in instances where they were not mobile and then the test was conducted at 24 h after birth.

Kids were tested for ability to discriminate their own dam from an alien dam [81] with modifications as noted by Gotardo *et al.* [29]. The test was performed at 12 h after birth. If kids did not successfully leave the start box, or did not complete the test by moving toward one dam or the other, the test was repeated at 24 h after birth. The alien dam used in each test was one that had recently given birth (within 5 days after delivery). Kids were not allowed to nurse for 120 min before the test. After this period they were placed into the test pen start box and allowed to choose either the mother on one side or the alien dam on the other side (Figure 1). Kids were allowed a maximum of 5 min to complete this test. Several variables were recorded during this test: (1) time to exit start box; (2) time to arrival (*i.e.*, physical touching) at either dam or alien dam and (3) choice (mother or alien dam). The test was performed twice; after the first test, dam positions were switched and the test repeated immediately.

#### 5.3.3. Kid Tests of Movements

**Figure 2:**
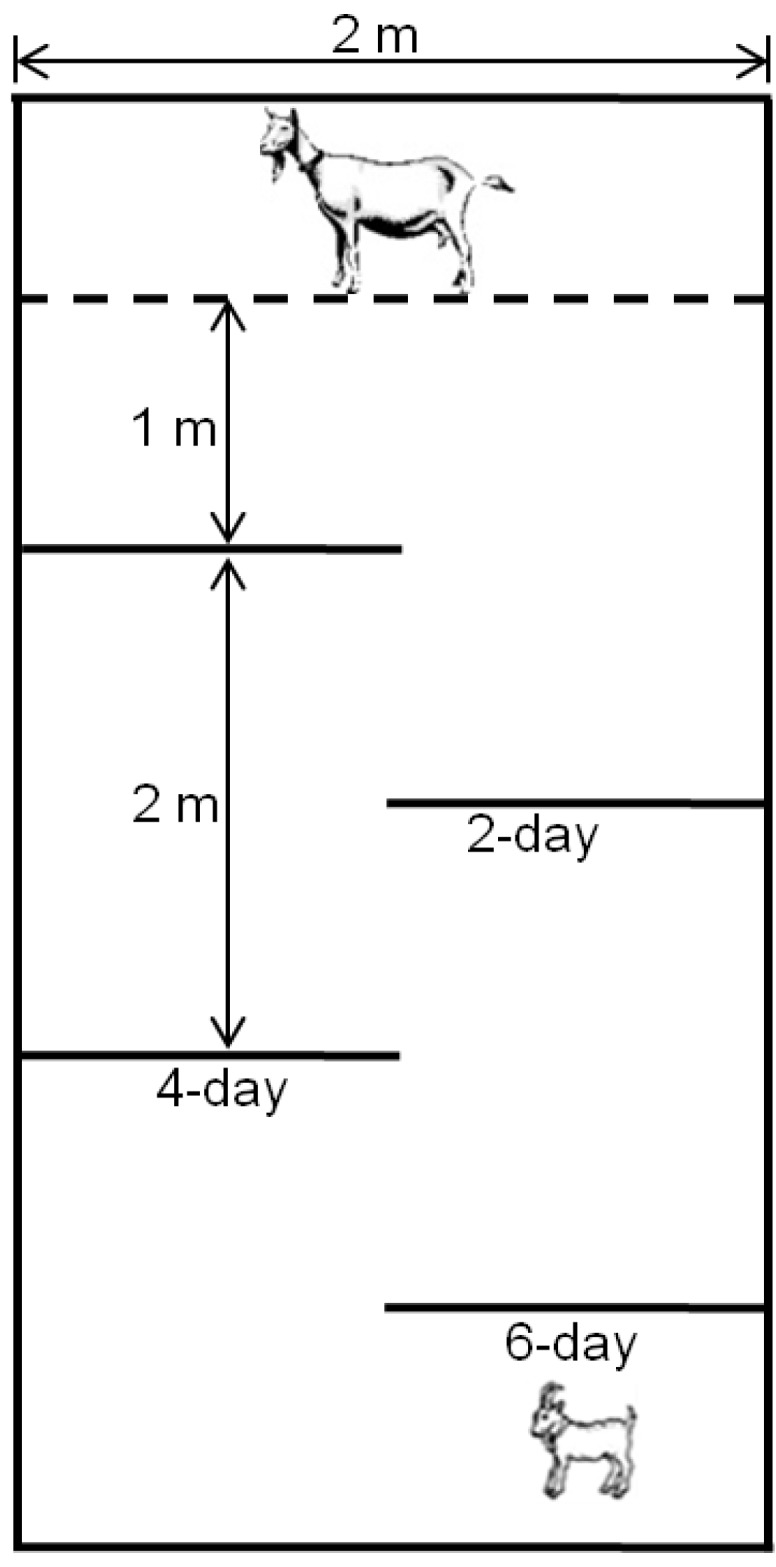
Schematic diagram of a maze used to test kids ability to navigate a progressively more challenging maze to reach its dam on days two, four, and six postpartum. Additional solid partitions as shown were added for the four- and six-day tests, respectively. The dam was tethered at the end of the maze.

Kids were tested at two, four, and six days in a progressive maze with incrementally increasing difficulty (Figure 2) as outlined in Gotardo *et al.* [29]. At exactly 48 h after parturition, kids were placed into a simple maze with the dam secured at the exit of the maze at a distance of 1 m. The 2-day maze had two barriers, the 4-day test had three barriers, and the 6-day maze had four barriers which the kids needed to navigate to access their mothers. Each kid was run three times in each test, with the maximum time allowed for each run 3 min. Variables recorded were (1) time to leave the starting position, and (2) time to traverse the maze and reach the dam.

#### 5.3.4. Hebb-Williams Mazes

**Figure 3:**
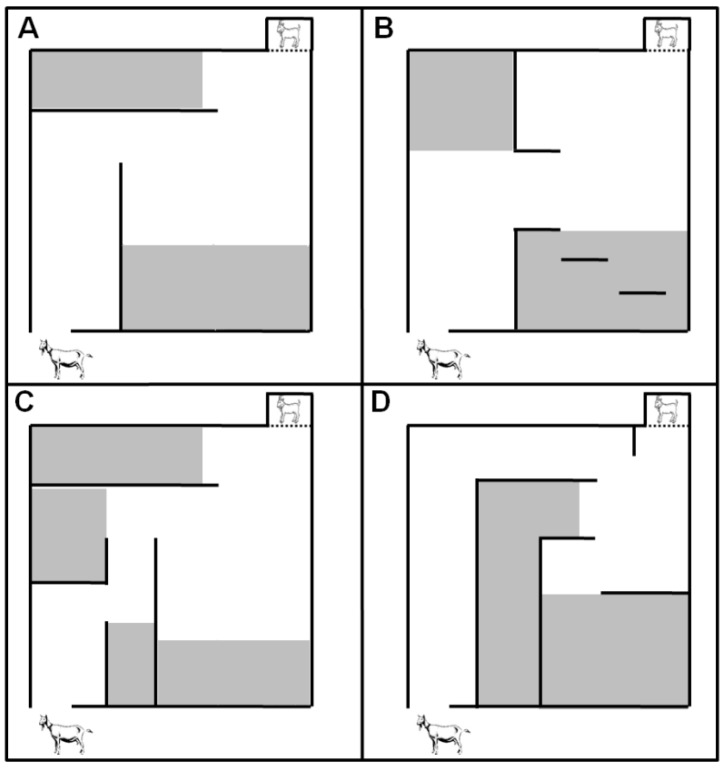
Schematic diagram of Hebb-Williams mazes used to test kids’ ability to navigate a maze to reach their mothers at two, four, and six weeks of age. Blind alleys are shown in gray; kids entering these alleys were considered errors. (**A**) Hebb-Williams A; (**B**) Hebb-Willians B; (**C**) Hebb-Willians C; (**D**) Hebb-Willians D.

The spatial abilities of kids in the Hebb-Williams maze [71] were examined at two, four, and six weeks of age in four different mazes (Figure 3). Each day, kids performed three consecutive runs in each of the four mazes. Before the tests kids were deprived of contact with their mothers for four hours. Immediately before the start of the tests, the mother was put in touch with its kid for a few seconds, to allow the dam to make physical contact and to begin to vocalize for her offspring. Similarly, this contact allowed the kid to know that the mother was nearby and served to motivate the kid to traverse the maze to reach the dam. The maximum time allowed to perform the correct route for each run was 5 min.

The following variables were recorded using a video camera for later viewing: (1) time to leave the starting position; (2) time to traverse the maze and reach the dam (*i.e.*, physical touching); and (3) number of errors (*i.e.*, wrong turns into blind alleys).

### 5.4. Statistical Analysis

For body weight gain during pregnancy, birth weight and variables for 2 h after birth, the Bartlett test was used to determine whether the data were normally and homogeneously distributed. A one-way ANOVA with Dunnett’s post hoc test was used to compare the groups for each variable, using SAS software (Version 9.2; SAS Institute, Cary, NC, USA, 2008). Two statistical tests were used to examine kids’ choice of own dam or alien dam in the discrimination test. A binomial test was done using a probability of 0.5 to test if choices were different from random selection; additionally a 2 × 2 chi square test of independence was also done to determine if control and IC kids groups differed in their number of incorrect choices. The times for kids in the dam-alien goat discrimination test, kid tests of movement, and Hebb-Williams mazes were analyzed using a mixed linear model (Proc Mixed) in SAS software (Version 9.2; SAS Institute, Cary, NC, USA, 2008)). The model included treatment, animals nested with treatment, and days and runs within days as repeated measures. The number of mistakes made by animals in the “Hebb-Williams” maze was analyzed in the same way, but taking into account the amount of error in each maze of each run. In all cases, the probability of significant differences was set at α = 0.05.

## Figures and Tables

**Table 1:** Reproductive parameters in goats treated with *I. carnea* from day 27 of pregnancy to term, and birth weight of their kids.

Parameters	Control (6) ^a^	*I. carnea* (g/kg Body Weight)		
		1.0 (6)	3.0 (6)	5.0 (6)
BWG ^b^ in pregnancy ^c^	6.1 ± 1.7	4.2 ± 2.3	6.5 ± 2.1	5.8 ± 1.6
Length of gestation (days) ^c^	149.1 ±0.7 (6) ^d^	150.1 ± 0.9 (6)	148.3 ± 1.8 (5)	151.2 ± 0.7 (5)
Abortion	0	0	1	1
Live kids	9	6	6	8
Male/Female	6/3	2/4	4/2	2/6
Twins	3	0	1	3
Kids body weight (kg) at birth ^c^	3.1 ± 0.1	3.5 ± 0.10	3.2 ± 0.2	2.9 ± 0.2

^a^ Number of goats; ^b^ Bodyweight gain (kg); ^c^ Mean ± SEM; ^d^ Number of deliveries.

**Table 2:** Dam behavior parameters during the first two hours after birth (mean ± SEM).

Variable ^a^	Period (min)	Control (6) ^b^	*I. carnea* (g/kg Body Weight)		
			1 (6)	3 (5)	5 (5)
Attention to kid (min)	0–60	56.5 ± 1.3	51.6 ± 3.4	33.6 ± 3.9 *	35.8 ± 3.0 *
	60–120	52.0 ± 2.5	47.0 ± 4.4	33.6 ± 2.2 *	31.2 ± 5.1 *
Sniffing/Licking(frequency)	0–15	56.2 ± 2.1	47.6 ± 3.6	25.2 ± 4.1 *	29.4 ± 1.4 *
	15–30	42.6 ± 1.5	41.4 ± 1.7	32.6 ± 4.5	25.6 ± 2.5 *
	30–60	57.2 ± 2.2	47.2 ± 3.6	26.2 ± 4.8 *	23.4 ± 2.5 *
	60–120	36.2 ± 2.2	28.6 ± 2.6	38.8 ± 3.6	24.2 ± 4.5

^a^ Dam behavior was videotaped with a low-light camcorder, and observations recorded for the various periods. Definitions are provided in the text; ^b^ Number of dams. * *p* < 0.05 compared with controls.

**Table 3:** Kids’ latencies (s; mean ± SEM) to first attempt to stand and to successfully stand, and time standing (min; mean ± SEM) during the first two hours after birth.

Variable ^a^	Control (9) ^b^	*I. carnea* (g/kg Body Weight)		
		1 (6)	3 (6)	5 (8)
First attempt to stand	95.0 ± 16.5	85.8 ± 26.8	655.0 ± 154.7 *	832.0 ± 439.4 *
Successfully stand	466.7 ± 88.5	1220.7 ± 420.3	2254.4 ± 1621.3	3668.0 ± 2018.8 *
Time standing	39.7 ± 3.3	27.8 ± 6.6	17.6 ± 5.4 *	2.2 ± 1.9 *

^a^ Kid behavior was videotaped with a low-light camcorder, and observations recorded for the various periods. Definitions are provided in the text; ^b^ Number of kids. * *p* < 0.05 compared with controls.

**Table 4:** Kids’ behavior variables (mean frequency ± SEM/Time period) during the first two hours after birth.

Variable ^a^	Control (9) ^b^	*I. carnea* (g/kg Body Weight)			Control (9)	*I. carnea* (g/kg Body Weight)		
		1 (6)	3 (6)	5 (8)		1 (6)	3 (6)	5 (8)
	0–15 min				15–30 min			
Crawl	2.3 ± 0.8	8.8 ± 1.8	9.8 ± 1.6 *	13.2 ± 2.7 *	0.5 ± 0.5	2.8 ± 0.5	3.4 ± 1.2	3.0 ± 2.3
Head up	0.6 ± 0.3	2.2 ± 0.7	2.4 ± 0.9	0.6 ± 0.4	0.2 ± 0.2	2.4 ± 1.5	0.4 ± 0.4	0
Attempt to stand	16.3 ± 1.2	16.4 ± 4.9	3.8 ± 2.3 *	4.6 ± 1.9 *	4 ± 1.4	10 ± 2	5.8 ± 0.9	6.2 ± 4.0
Stand up	5.2 ± 1.4	0.4 ± 0.2 *	0 *	0.6 ± 0.6 *	4.6 ± 1.1	2.8 ± 1.3	3.4 ± 2.5	1.2 ± 1.2
Nuzzle dam’s front half	4.0 ± 1.5	0 *	0 *	0 *	18.0 ± 2.6	3.2 ± 1.8 *	1.2 ± 1.2 *	0 *
Nuzzle dam’s rear half	3.5 ± 0.8	0 *	0 *	0 *	22.5 ± 1.3	2.4 ± 1.5 *	3.2 ± 3.2 *	0 *
Suckle	0.3 ± 0.2	0	0	0	6.0 ± 1.0	1.2 ± 0.6 *	0 *	0 *
Walk to dam	1.2 ± 0.5	0 *	0 *	0 *	6.0 ± 1.7	0.8 ± 0.4 *	0.4 ± 0.4 *	0 *
Walk away from dam	0	0	0	0	0.2 ± 0.2	0.4 ± 0.4	0.4 ± 0.2	0.4 ± 0.4
	**30–60 min**				**60–120 min**			
Crawl	0	1.6 ± 1.0	2.4 ± 1.0	5.4 ± 3.1	0	0.2 ± 0.2	2.2 ± 0.4	3.2 ± 2.7
Head up	0	0	0.6 ± 0.4	0.8 ± 0.8	0	0.2 ± 0.2	0.2 ± 0.2	2.0 ± 1.3
Attempt to stand	0.3 ± 0.3	6.0 ± 4.3	2.2 ± 0.4	6.2 ± 2.5	0	1.6 ± 0.7	5.6 ± 3.9	3.0 ± 2.7
Stand up	5.8 ± 0.9	4.0 ± 1.3	5.0 ± 2.5	1.4 ± 0.9	5.7 ± 1.7	5.4 ± 1.8	9.4 ± 2	12.4 ± 2.5
Nuzzle dam’s front half	3.8 ± 0.7	4.6 ± 1.2	4.0 ± 1.6	0.4 ± 0.4	3.3 ± 0.6	4.0 ± 1.2	6.2 ± 1.3	2.2 ± 0.9
Nuzzle dam’s rear half	9.0 ± 1.0	6.2 ± 2.6	5.8 ± 3.3	0.4 ± 0.4 *	5.5 ± 1.2	15.4 ± 5.2	9.0 ± 1.9	4.6 ± 2.1
Suckle	12.0 ± 1.8	6.6 ± 2.2 *	0.4 ± 0.4 *	0 *	6.5 ± 0.6	3.8 ± 2.6	1.4 ± 0.9	1.8 ± 1.3
Walk to dam	8.3 ± 2.2	1.0 ± 0.4 *	1.0 ± 0.6 *	0.4 ± 0.4 *	10.0 ± 2.8	1.6 ± 0.5 *	1.2 ± 0.7 *	2.2 ± 1.2 *
Walk away from dam	0.8 ± 0.9	0.2 ± 0.4	1.2 ± 1.1	1.0 ± 2.2	0.3 ± 0.5	2.0 ± 2.0	2.0 ± 1.6	2.4 ± 2.3

^a^ Kid behavior was videotaped with a low-light camcorder, and observations recorded for the various periods. Definitions are provided in the text; ^b^ Number of kids. * *p* < 0.05 compared with controls.

**Table 5:** Discrimination of kids for own or an alien dam (mean ± SEM) at 12 h after birth.

Variable	Run	Variable	Control (9) ^a^	*I. carnea* (g/kg Body Weight)		
				1 (6)	3 (6)	5 (8)
Time	1	Exit ^b^	17.6 ± 3.2	138.9 ± 44.1 *	172.7 ± 39.5 *	152.9 ± 32.1 *
		Arrival ^c^	46.8 ± 7.3	155.4 ± 110.4 *	189.0 ± 35.9 *	188.8 ± 27.5 *
	2	Exit	16.3 ± 4.8	79.4 ± 37.1	252.1 ± 32.3 *	175.8 ± 33.8 *
		Arrival	32.8 ± 6.2	99.1 ± 34.9	259.6 ± 30.6 *	187.4 ± 31.1 *
Incorrect choices ^d^	1	-	0/18	6/12 **	8/12 **	13/16 **
	2	-	2/18	8/12 **	11/12 **	12/16 **

Kids were each given two tests (*i.e.*, runs) with the alien and own dam reversed in position from test 1 to test 2. ^a^ Number of kids; ^b^ Time (s) for kids to leave the start box; ^c^ Time (s) for kids to complete the maze, and touch either their own dam or the alien dam; ^d^ Incorrect choice of alien dam/number of attempts by kids. * *p* < 0.05 compared with controls. ** *p* < 0.001 compared with controls.

**Table 6:** Kids performance in a progressive maze (mean ± SEM) on postpartum days 2, 4, and 6.

Day	Control (9) ^a^		*I. carnea* (g/kg Body Weight)					
			1 (6)		3 (6)		5 (8)	
	Leave ^b^	Arrive ^c^	Leave	Arrive	Leave	Arrive	Leave	Arrive
2	13.3 ± 3.7	17.9 ± 4.0	54.7 ± 17.2	59.2 ± 16.6	72.1 ± 19.2 *	76.3 ± 18.5 *	68.4 ± 14.6 *	78.5 ± 14.1 *
4	9.5 ± 2.2	20.5 ± 3.4	17.3 ± 7.3	29.3 ± 9.2	93.2 ± 21.1 *	99.5 ± 19.7 *	49.0 ± 13.8 *	61.8 ± 12.9 *
6	5.1 ± 0.9	17.8 ± 2.0	3.6 ± 0.6	20.0 ± 8.6	57.2 ± 17.3 *	71.2 ± 17.9 *	39.3 ± 12.2	49.5 ± 11.9

The maze had two barriers for day 2, three for day 4, and four for day 6. ^a^ Number of kids; ^b^ Time (s) for kids to leave the starting point in the maze after being placed into position; ^c^ Time (s) for kids to complete the maze. * *p* < 0.05 compared with controls.

**Table 7:** Total number of errors of the kids in the Hebb-Williams mazes A, B, C and D (HW-A; HW-B; HW-C; and HW-D; mean ± SEM) in postpartum weeks 2, 4, and 6.

Maze	Control (9) ^a^	*I. carnea* (g/kg Body Weight)		
		1 (6)	3 (6)	5 (8)
HW-A	0.17 ± 0.09 ^b^	0.44 ± 0.14	0.53 ± 0.18	0.84 ± 0.21 *
HW-B	0.2 ± 0.07	0.16 ± 0.06	0.42 ± 0.12	0.46 ± 0.12
HW-C	0.17 ± 0.05	0.56 ± 0.11	0.29 ± 0.09	0.78 ± 0.14 *
HW-D	1.72 ± 0.32	2 ± 0.28	1.93 ± 0.29	2. 02 ± 0.26

^a^ Number of kids; ^b^ Summation errors of each run in weeks 6, 8 and 10 of age. * *p* < 0.05 compared with controls.

**Table 8:** Kids’ performance in a four different Hebb-Williams mazes A, B, C and D (HW-A; HW-B; HW-C; and HW-D; mean ± SEM) in postpartum weeks 2, 4, and 6.

Maze/Week	Control (9) ^a^		*I. carnea* (g/kg Body Weight)					
			1 (6)		3 (6)		5 (8)	
	Leave ^b^	Arrive ^c^	Leave	Arrive	Leave	Arrive	Leave	Arrive
HW-A								
Week 2	3.4 ± 1.2	16.4 ± 3.5	3.1 ± 0.6	99.5 ± 31.0 *	5.7 ± 1.5	155.8 ± 35.0 *	8.1 ± 2.4 *	158.8 ± 24.4 *
Week 4	1.8 ± 0.3	9.2 ± 1.6	8.4 ± 4.7 *	52.6 ± 26.1	2.2 ± 0.5	14.2 ± 3.9	2.1 ± 0.2	9.8 ± 1.4
Week 6	1.7 ± 0.3	8.3 ± 0.8	3.2 ± 0.4	13.1 ± 3.1	1.6 ± 0.1	14.3 ± 4.0	5.1 ± 1.0	31.1 ± 6.7
HW-B								
Week 2	2.6 ± 0.4	24.6 ± 6.7	4.3 ± 1.1	29.5 ± 10.7	2.0 ± 0.4	61.1 ± 26.1	4.0 ± 1.1	84.8 ± 23.2 *
Week 4	1.8 ± 0.2	9.0 ± 1.1	2.7 ± 0.4	21.7 ± 8.5	2.1 ± 0.1	22.2 ± 9.9	2.7 ± 0.3	16.9 ± 3.8
Week 6	1.5 ± 0.1	7.5 ± 0.8	2.0 ± 0.3	9.9 ± 1.4	2.0 ± 0.2	12.2 ± 3.1	3.9 ± 0.8 *	39.6 ± 8.8 *
HW-C								
Week 2	2.1 ± 0.3	15.4 ± 2.3	5.0 ± 1.9	23.0 ± 8.7	1.7 ± 0.4	26.1 ± 12.7	6.8 ± 3.2 *	50.7 ± 17.7
Week 4	1.7 ± 0.1	8.1 ± 0.7	1.7 ± 0.3	24.2 ± 6.7	4.8 ± 2.1	22.9 ± 8.5	4.8 ± 0.8	28.4 ± 4.8
Week 6	1.8 ± 0.2	7.7 ± 0.6	2.2 ± 0.4	14.9 ± 2.7	1.8 ± 0.2	8.5 ± 1.0	5.6 ± 1.0	30.6 ± 6.4 *
HW-D								
Week 2	2.6 ± 0.4	68.2 ± 8.3	6.2 ± 2.3	143.8 ± 32.8	3.1 ± 0.9	232.9 ± 25.8 *	4.9 ± 1.3	157.8 ± 23.7 *
Week 4	1.5 ± 0.1	27.3 ± 3.6	2.9 ± 0.8	77.8 ± 11.8	2.9 ± 0.7	118.7 ± 21.9 *	5.2 ± 0.9	92.6 ± 14.7 *
Week 6	1.6 ± 0.1	23.2 ± 2.9	3.7 ± 0.9	56.4 ± 12.1 *	3.1 ± 1.0	48.2 ± 14.6	7.5 ± 1.9 *	58.7 ± 9.8 *

^a^ Number of kids; ^b^ Time (s) for kids to leave the starting point in the maze after being placed into position; ^c^ Time (s) for kids to complete the maze. * *p* < 0.05 compared with controls.
